# Smoking and Major Depressive Disorder in Chinese Women

**DOI:** 10.1371/journal.pone.0106287

**Published:** 2014-09-02

**Authors:** Qiang He, Lei Yang, Shenxun Shi, Jingfang Gao, Ming Tao, Kerang Zhang, Chengge Gao, Lijun Yang, Kan Li, Jianguo Shi, Gang Wang, Lanfen Liu, Jinbei Zhang, Bo Du, Guoqing Jiang, Jianhua Shen, Zhen Zhang, Wei Liang, Jing Sun, Jian Hu, Tiebang Liu, Xueyi Wang, Guodong Miao, Huaqing Meng, Yi Li, Chunmei Hu, Yi Li, Guoping Huang, Gongying Li, Baowei Ha, Hong Deng, Qiyi Mei, Hui Zhong, Shugui Gao, Hong Sang, Yutang Zhang, Xiang Fang, Fengyu Yu, Donglin Yang, Tieqiao Liu, Yunchun Chen, Xiaohong Hong, Wenyuan Wu, Guibing Chen, Min Cai, Yan Song, Jiyang Pan, Jicheng Dong, Runde Pan, Wei Zhang, Zhenming Shen, Zhengrong Liu, Danhua Gu, Xiaoping Wang, Ying Liu, Xiaojuan Liu, Qiwen Zhang, Yihan Li, Yiping Chen, Kenneth S. Kendler, Xumei Wang, Youhui Li, Jonathan Flint

**Affiliations:** 1 ShengJing Hospital of China Medical University, Heping District, Shenyang, Liaoning, P. R. China; 2 The First Affiliated Hospital of Zhengzhou University, Zhengzhou, Henan, P. R. China; 3 Shanghai Mental Health Center, Shanghai, P. R. China; 4 Huashan Hospital of Fudan University, Shanghai, P. R. China; 5 Chinese Traditional Hospital of Zhejiang, Hangzhou, Zhejiang, P. R. China; 6 Xinhua Hospital of Zhejiang Province, Hangzhou, Zhejiang, P. R. China; 7 No. 1 Hospital of Shanxi Medical University, Taiyuan, Shanxi, P. R. China; 8 No. 1 Hospital of Medical College of Xian Jiaotong University, Xian, Shaanxi, P. R. China; 9 Jilin Brain Hospital, Siping, Jilin, P. R. China; 10 Mental Hospital of Jiangxi Province, Nanchang, Jiangxi, P. R. China; 11 Xian Mental Health Center, New Qujiang District, Xian, Shaanxi, P. R. China; 12 Beijing Anding Hospital of Capital University of Medical Sciences, Deshengmen wai, Xicheng District, Beijing, P. R. China; 13 Shandong Mental Health Center, Jinan, Shandong, P. R. China; 14 No. 3 Hospital of Sun Yat-sen University, Tianhe District, Guangzhou, Guangdong, P. R. China; 15 Hebei Mental Health Center, Baoding, Hebei, P. R. China; 16 Chongqing Mental Health Center, Jiangbei District, Chongqing, P. R. China; 17 Tianjin Anding Hospital, Hexi District, Tianjin, P. R. China; 18 No. 4 Hospital of Jiangsu University, Zhenjiang, Jiangsu, P. R. China; 19 Psychiatric Hospital of Henan Province, Xinxiang, Henan, P. R. China; 20 Nanjing Brain Hospital, Nanjing, Jiangsu, P. R. China; 21 Harbin Medical University, Nangang District, Haerbin, Heilongjiang, P. R. China; 22 Shenzhen Kang Ning Hospital, Luohu District, Shenzhen, Guangdong, P. R. China; 23 First Hospital of Hebei Medical University, Shijiazhuang, Hebei, P. R. China; 24 Guangzhou Brain Hospital (Guangzhou Psychiatric Hospital), Liwan District, Guangzhou, Guangdong, P. R. China; 25 No. 1 Hospital of Chongqing Medical University, Yuanjiagang, Yuzhong District, Chongqing, P. R. China; 26 Dalian No. 7 Hospital, Ganjingzi District, Dalian, Liaoning, P. R. China; 27 No. 3 Hospital of Heilongjiang Province, Beian, Heilongjiang, P. R. China; 28 Wuhan Mental Health Center, Wuhan, Hubei, P. R. China; 29 Sichuan Mental Health Center, Mianyang, Sichuan, P. R. China; 30 Mental Health Institute of Jining Medical College, Dai Zhuang, Bei Jiao, Jining, Shandong, P. R. China; 31 Liaocheng No. 4 Hospital, Liaocheng, Shandong, P. R. China; 32 Mental Health Center of West China Hospital of Sichuan University, Wuhou District, Chengdu, Sichuan, P. R. China; 33 Suzhou Guangji Hospital, Suzhou, Jiangsu, P. R. China; 34 Anhui Mental Health Center, Hefei, Anhui, P. R. China; 35 Ningbo Kang Ning Hospital, Zhenhai District, Ningbo, Zhejiang, P. R. China; 36 Changchun Mental Hospital, Changchun, Jilin, P. R. China; 37 No. 2 Hospital of Lanzhou University, Lanzhou, Gansu, P. R. China; 38 Fuzhou Psychiatric Hospital, Cangshan District, Fuzhou, Fujian, P. R. China; 39 Harbin No. 1 Special Hospital, Haerbin, Heilongjiang, P. R. China; 40 Jining Psychiatric Hospital, North Dai Zhuang, Rencheng District, Jining, Shandong, P. R. China; 41 No. 2 Xiangya Hospital of Zhongnan University, Furong District, Changsha, Hunan, P. R. China; 42 Xijing Hospital of No. 4 Military Medical University, Xian, Shaanxi, P. R. China; 43 Mental Health Center of Shantou University, Shantou, Guangdong, P. R. China; 44 Tongji University Hospital, Shanghai, P. R. China; 45 Huaian No. 3 Hospital, Huaian, Jiangsu, P. R. China; 46 Huzhou No. 3 Hospital, Huzhou, Zhejiang, P. R. China; 47 Mudanjiang Psychiatric Hospital of Heilongjiang Province, Xinglong, Mudanjiang, Heilongjiang, P. R. China; 48 No. 1 Hospital of Jinan University, Guangzhou, Guangdong, P. R. China; 49 Qingdao Mental Health Center, Shibei District, Qingdao, Shandong, P. R. China; 50 Guangxi Longquanshan Hospital, Yufeng District, Liuzhou, P. R. China; 51 Daqing No. 3 Hospital of Heilongjiang Province, Ranghulu district, Daqing, Heilongjiang, P. R. China; 52 Tangshan No. 5 Hospital, Lunan District, Tangshan, Hebei, P. R. China; 53 Anshan Psychiatric Rehabilitation Hospital, Lishan District, Anshan, Liaoning, P. R. China; 54 Weihai Mental Health Center, ETDZ, Weihai, Shandong, P. R. China; 55 Renmin Hospital of Wuhan University, Wuchang District, Wuhan, Hubei, P. R. China; 56 The First Hospital of China Medical University, Heping District, Shenyang, Liaoning, P. R. China; 57 Tianjin First Center Hospital, Hedong District, Tianjin, P. R. China; 58 Hainan Anning Hospital, Haikou, Hainan, P. R. China; 59 Wellcome Trust Centre for Human Genetics, Oxford, United Kingdom; 60 Clinical Trial Service Unit, Richard Doll Building, Oxford, United Kingdom; 61 Virginia Institute for Psychiatric and Behavioral Genetics, Department of Psychiatry, Virginia Commonwealth University, Richmond, Virginia, United States of America; University of Iowa Hospitals & Clinics, United States of America

## Abstract

**Objective:**

To investigate the risk factors that contribute to smoking in female patients with major depressive disorder (MDD) and the clinical features in depressed smokers.

**Methods:**

We examined the smoking status and clinical features in 6120 Han Chinese women with MDD (DSM-IV) between 30 and 60 years of age across China. Logistic regression was used to determine the association between clinical features of MDD and smoking status and between risk factors for MDD and smoking status.

**Results:**

Among the recurrent MDD patients there were 216(3.6%) current smokers, 117 (2.0%) former smokers and 333(5.6%) lifetime smokers. Lifetime smokers had a slightly more severe illness, characterized by more episodes, longer duration, more comorbid illness (panic and phobias), with more DSM-IV A criteria and reported more symptoms of fatigue and suicidal ideation or attempts than never smokers. Some known risk factors for MDD were also differentially represented among smokers compared to non-smokers. Smokers reported more stressful life events, were more likely to report childhood sexual abuse, had higher levels of neuroticism and an increased rate of familial MDD. Only neuroticism was significantly related to nicotine dependence.

**Conclusions:**

Although depressed women smokers experience more severe illness, smoking rates remain low in MDD patients. Family history of MDD and environmental factors contribute to lifetime smoking in Chinese women, consistent with the hypothesis that the association of smoking and depression may be caused by common underlying factors.

## Introduction

Currently smoking remains the single most important and preventable cause of premature female death in some developed countries (such as the USA, the UK) [1]. Up to one third of female deaths between the ages of 35 to 69 can be attributed to smoking related factors [1]. However, smoking is still regarded as the male problem in many countries. Including China, where female smoking is not paid enough attention. This is due primarily to one reason: cigarette smoking prevalence among women in China is quite low compared to male, and the health consequences for women will not have started to emerge. In 1996 Yang et al recruited 128,766 participants in China and the prevalence rate for ever smokers was 4.2% for female and 66.9% for male [2]. In many countries religion, culture and lower socioeconomic status are the main factors that explain why women smoke less than men. While these factors protect women from smoking, as their influence wanes, the rates of female smoking are likely to increase [3]. The main diseases caused by smoking are cancers (especially lung cancer), heart disease and chronic bronchitis. These smoking related diseases affect both sexes equally, however smoking also causes specific impairments to women: (i) for women taking oral contraception the risk of both heart disease and stroke increases substantially; (ii) the risk of cervical cancer increases two fold; (iii) there are adverse affects on the female reproductive system, including dysmenorrhea, decreased fertility and early menopause [4].

Many studies (including cross-sectional and longitudinal) indicate an association between smoking (or nicotine dependence) and MDD [5], [6], [7], [8], [9], [10]. There are two explanations for this association: (i) smoking and MDD have the same potential risk factors (genetic or environmental factors) [5], [11]; (ii) there is a causal relation between smoking and MDD: MDD leads to smoking [12], [13], [14] or smoking leads to MDD[10], [14], [15], [16], [17].

The main aim of our research is to investigate in a large sample of Chinese women with recurrent MDD: (i) the differences in clinical features between depressed smokers and depressed non-smokers; (ii) the risk factors that contribute to smoking in female patients with MDD.

## Methods

### Samples

Data for the present study draw upon the China, Oxford and VCU Experimental Research on Genetic Epidemiology (CONVERGE) study of MDD. Analyses were based on a total of 6120 cases recruited from 53 provincial mental health centers and psychiatric departments of general medical hospitals in 41 cities in 19 provinces. All cases were female and had four Han Chinese grandparents. Cases were excluded if they had a pre-existing history of bipolar disorder, any type of psychosis or mental retardation. Cases were aged between 30 and 60, had two or more episodes of MDD, with the first episode occurring between 14 and 50 and had not abused drug or alcohol before the first episode of MDD. All subjects were interviewed using a computerized assessment system, which lasted on average two hours for a case. All interviewers were trained by the CONVERGE team for a minimum of one week in the use of the interview. The interview includes assessment of psychopathology, demographic and personal characteristics, and psychosocial functioning.

### Ethics Statement

The study protocol was approved centrally by the Ethical Review Board of Oxford University (Oxford Tropical Research Ethics Committee) and the ethics committees in all participating hospitals in China. Major psychotic illness was an exclusion criterion. All interviewers were mental health professionals who are well able to judge decisional capacity. The study posed minimal risk (an interview and saliva sample). All participants provided their written informed consents.

### Measures

The diagnoses of depressive (Dysthymia and Major Depressive Disorder) and anxiety disorders (Generalized Anxiety Disorder, Panic Disorder with or without Agoraphobia) were established with the Composite International Diagnostic Interview (CIDI) (WHO lifetime version 2.1; Chinese version), which classifies diagnoses according to the Diagnostic and Statistical Manual of Mental Disorders (DSM-IV) criteria [18].

The interview was originally translated into Mandarin by a team of psychiatrists in Shanghai Mental Health Centre with the translation reviewed and modified by members of the CONVERGE team.

Smoking status: Never smokers were those who had never smoked as many as one cigarette a day or equivalent for at least 1 month. Current smokers were persons who had ever smoked an average of at least one cigarette a day for at least 1 month and who were still smoking. Former smokers were those who had ever smoked an average of at least one cigarette a day for at least 1 month and who were not smoking currently. Both current and former smokers are lifetime smokers. For the lifetime smokers the Fagerstrom Test for Nicotine Dependence (FTND) scores were assessed at the time during their life when they smoked most heavily [19]. If the score of FTND > = 5, the smokers are defined as nicotine dependence smokers(ND smokers). The others were classified as non-ND smokers.

Phobias, divided into five subtypes (animal, situational, social, blood-injury and agoraphobia) were diagnosed using an adaptation of DSM-III criteria requiring one or more unreasonable fears, including fears of different animals, social phobia and agoraphobia that objectively interfered with the respondent's life. The section on the assessment of phobias was translated by the CONVERGE team from the interview used in the Virginia Adult Twin Study of Psychiatric and Substance Use Disorders (VATSPUD) [20]. Age at onset of MDD was assessed retrospectively and defined as the age at which the first manifestation of MDD occurred, as reported by the participants. Number of DSM-IV A criteria is regarded as the number of the symptoms in the worst MDD episode. Scores from 5 to 9.

Additional information was collected using instruments employed from VATSPSUD, translated and reviewed for accuracy by members of the CONVERGE team. The history of lifetime major depression in the parents and siblings was assessed using the Family History Research Diagnostic criteria [21]. A family history of MDD refers to patients who had at least one first-degree relative with MDD. The stressful life events (SLE) section, also developed for the VATSPSUD study, assessed 16 traumatic lifetime events and the age at their occurrence. SLE scores are from 0 to 16. The childhood sexual abuse (CSA) was a shortened version of the detailed module used in the VATSPSUD study, which was in turn based on the instrument developed by Martin et al [22] and included three forms of CSA: nongenital, genital, and intercourse. Any form of CSA was scored as 1. Absence of CSA was scored 0. Neuroticism was measured with the 23-item Eysenck Personality Questionnaire [23], an established instrument for measuring neuroticism. Neuroticism scores are from 0 to 23. Parent–child relationships were measured with the 16-item Parental Bonding Instrument (PBI) modified by Kendler [24] based on Parker's original 25-item instrument [25]. Three factors were extracted from these 16 items and labeled warmth, protectiveness and authoritarianism. Premenstrual symptoms (PMS) were assessed from four questions about the psychological aspects of the experience [26]. Answers, reported as “a lot”, “some”, “little” or “not at all”, were scored numerically between 4 (“a lot”) and 1 (“not at all”) and a total score obtained for each subject.

All interviews were carried out using a computerized system developed in house in Oxford and called SysQ. Skip patterns were built into SysQ. Interviews were administered by trained interviewers and entered offline in real time onto SysQ, which is installed on laptops. The backup file, together with an audio recording of the entire interview, were uploaded to a designated server currently maintained in Beijing by a service provider. All the uploaded files in the Beijing server were then transferred to an Oxford server quarterly.

### Statistical analysis

Sociodemographic and clinical characteristics of the sample were analysed. For continuous variables, independent Student's t tests and U tests were performed and for categorical variables, Pearson's χ2 were calculated. All characteristics of individuals with lifetime smoking versus never smoking were assessed by logistic regression, with smoking as the dependent variable (0 = never smoking and 1 = lifetime smoking). Associations between variables were expressed as odds ratios (OR) and 95% confidence intervals (95% CI). SPSS 16.0 for Windows was used in data analysis.

## Results

We obtained 6120 cases of recurrent MDD from 53 hospitals in China. Among them 5996 (98.0%) provided complete data and were included in the analysis. 3.6% of our cases (n = 216) were current smokers; 2.0% (n = 117) were former smokers; 5.6% (n = 333) were lifetime smokers; 6.6% (n = 393) reported smoking sometime (but not regularly) in their lives and 94.4% (n = 5663) were never smokers. Women who smoked were on average two years younger than those who did not (mean age of the smokers was 42.5 years compared to 44.6 years for never smokers, P = 0.0002, t = −3.75, df = 359.26). Most of the smoking patients (60.7%) reported taking up smoking before the first onset of MDD.

Table 1 shows the socio-economic characteristics of smokers and non-smokers among those with MDD. Smokers tend to have less education and are more likely to be unemployed or keeping house, but these differences do not reach statistical significance. However we noted a relatively large effect attributable to marital status. Being unmarried was associated with a highly significant increase in the likelihood of smoking (odds ratio = 2.920, 95%CI = 2.3–3.7, P = 8.8E-20).

**Table 1:** Socio-economic factors related to lifetime smoking.

	Lifetime Smokers N = 333 N(%)	Never smokers N = 5663 N(%)
**Education**		
Bachelor degree or higher	38(11.5)	725(12.8)
Adult schooling/Junior college	46(13.9)	713(12.6)
Senior middle school/Technical and vocational school	87(26.4)	1453(25.7)
Junior middle school	73(22.1)	1614(28.6)
Primary school and lower	86(26.1)	1146(20.3)
**Employment status**		
Full or part time work	101(30.6)	1823(32.3)
Pension/sickness benefits	37(11.2)	606(10.7)
Retired from a paid job	50(15.2)	1156(20.5)
Unemployed/keeping house/staying at home	125(37.9)	1725(30.5)
Other (e.g.student, permanently disabled)	17(5.2)	342(6.1)
**Social class**		
major and lesser professionals	42(12.7)	677(12.0)
Minor Professionals	116(35.2)	1890(33.5)
Skilled Manual Employees	42(12.7)	839(14.9)
Semi-Skilled and Unskilled Workers	81(24.5)	1337(23.7)
Other	49(14.8)	905(16.0)
**Marital status**		
Married	216(65.5)	4785(84.7)
Divorced/separated/widowed/never married	114(34.5)	865(15.3)

We next looked at the association between clinical features and smoking to determine if they distinguish between lifetime smokers and never smokers. We incorporated age as a covariate and show results in Table 2. Table 2 shows that the smokers have a slightly more severe illness, characterized by more episodes, longer duration, with more DSM-IV A criteria, though the effects are small. Correcting for multiple testing across all 23 variables described in tables 2 and 3, we obtain a 5% significance threshold of 0.002. This is exceeded by three results, namely those for association with the number of DSM A criteria, panic and situational phobia. However in Table 2 seven features are associated with significance less than 0.05, when only one would be expected by chance. We would expect that with a large sample, the additional associations would reach formal statistical significance.

**Table 2:** Clinical features and comorbid disorders associated with lifetime smoking, controlling for the effect of age.

Clinical feature	P-value	Odds ratio	95% CI
Age of onset	0.326	0.99	0.98–1.01
Number of episodes	0.061	1.01	1.00–1.02
Length of longest episode	0.012	1.00	1.00–1.01
Number of DSM A criteria	0.0005	1.28	1.11–1.47
Dysthymia	0.295	1.21	0.85–1.71
Melancholia	0.782	1.05	0.76–1.45
Panic	0.000041	2.08	1.46–2.94
GAD	0.841	0.97	0.75–1.26
Agoraphobia	0.015	1.46	1.08–1.99
Social phobia	0.042	1.40	1.01–1.93
Animal phobia	0.003	1.46	1.14–1.87
Blood phobia	0.100	1.27	0.95–1.69
Situational phobia	0.001	1.57	1.19–2.08

**Table 3:** Risk factors for MDD associated with lifetime smoking, controlling for the effect of age.

Phenotype		Lifetime smokers (n = 333)	Never smokers (n = 5663)	OR and 95% CI	P-value
Neuroticism (standardized)		0.26 (0.92)	−0.02 (1.002)	1.34(1.19–1.51)	1.99E-06
Childhood sexual abuse		63 (19.2%)	544 (9.7%)	2.24(1.68–2.98)	5.14E-07
Family history of MDD		109 (32.7%)	1463 (25.8%)	1.38(1.09–1.76)	0.008
Number of stressful life events		2.454 (2.2)	1.51 (1.631)	1.29(1.22–1.36)	8.88E-20
Perceived parenting	Warmth (maternal)	14.676 (5.6)	14.17 (5.1)	1.02(0.99–1.04)	0.13
	Warmth (paternal)	15.243 (5.4)	15.04 (5.1)	1.01(0.98–1.03)	0.58
	Authoritarianism (maternal)	8.96 (3.8)	8.86 (3.6)	1.01(0.97–1.04)	0.61
	Authoritarianism (paternal)	9.09 (3.6)	8.85 (3.5)	1.02(0.98–1.06)	0.38
	Protectiveness (maternal)	10.16 (3.2)	9.45 (2.9)	1.08(1.05–1.12)	0.000624
	Protectiveness (paternal)	9.70 (3.0)	9.12(2.8)	1.07(1.03–1.12)	0.00297

The largest effect was that for the number of DSM-IV A criteria, with an odds ratio of 1.28. We asked whether smokers reported any particular A criteria more frequently and found that only fatigue (OR = 1.94, 95%CI = 1.08–3.48, P = 0.027) and suicidal ideation or attempts (OR = 1.36, 95%CI = 1.02–1.80, P = 0.032) were over-represented.

Table 2 also shows an enrichment of comorbid anxiety disorders. We did not find that smokers were more likely to suffer dysthymia, melancholia or generalized anxiety disorder (GAD). However among the anxiety disorders, smokers suffered more phobias and were twice as likely to report panic compared to non-smokers.

We examined a series of known risk factors for MDD to see if these were differentially represented among smokers compared to non-smokers, after controlling for age. Table 3 shows results for neuroticism, family history of depression, stressful life events, childhood sexual abuse and perceived parenting. Smokers report more stressful life events, are more likely to report childhood sexual abuse, have higher levels of neuroticism and an increased rate of familial MDD. The largest effects were for childhood sexual abuse with OR>2.2. We found that only one of the three perceived parenting factors significantly differentiated smokers from non-smokers. Smokers reported higher rates of protectiveness (from both mother and father), although the effect was small (OR of 1.08).

Among smokers, we identified 165 women who we defined as nicotine dependent, based on an FTND score greater than 5 (49.6% of the total). Tables 4 and 5 show results for testing the relationship between clinical phenotypes and nicotine dependence. After correcting for multiple testing, none of the clinical features given in table 4 are significant. The smallest P-value we obtained was 0.041, for the number of DSM criteria. A Bonferroni corrected 5% threshold for testing 13 features is 0.004. Table 5 shows that there is a significant association between neuroticism and nicotine dependence. With a P-value of 0.0005 this survives correction even for testing all 23 variables (5% threshold of 0.002).

**Table 4:** Clinical features and comorbid disorders associated with nicotine dependency, non-ND smokers(n = 167) to ND smokers(n = 165), controlling for the effect of age.

Clinical feature	P-value	Odds ratio	95% CI
Age of onset	0.890	1.00	0.97–1.03
Number of episodes	0.108	1.02	1.00–1.03
Length of longest episode	0.929	1.00	0.998–1.002
Number of DSM A criteria	0.041	1.36	1.01–1.82
Dysthymia	0.676	0.87	0.45–1.68
Melancholia	0.666	1.15	0.61–2.19
Panic	0.148	1.65	0.84–3.24
GAD	0.187	0.713	0.43–1.18
Agoraphobia	0.048	1.84	1.01–3.38
Social phobia	0.133	1.62	0.86–3.05
Animal phobia	0.821	1.06	0.66–1.70
Blood phobia	0.384	0.78	0.45–1.36
Situational phobia	0.095	1.58	0.92–2.70

**Table 5:** Risk factors for MDD associated with nicotine dependency, controlling for the effect of age.

Phenotype		ND smokers (n = 165)	Non-ND smokers (n = 167)	OR and 95% CI	P-value
Neuroticism (standardized)		0.43 (0.905)	0.10(0.895)	1.58(1.22–2.05)	0.0005
Childhood sexual abuse		30 (18.4%)	34 (20.6%)	0.95(0.54–1.68)	0.864
Family history of MDD		54 (32.7%)	55 (32.9%)	1.031(0.65–1.64)	0.898
Number of stressful life events		2.65 (2.23)	2.25 (2.23)	1.10(0.99–1.23)	0.071
Perceived parenting	Warmth (maternal)	14.76 (5.60)	14.59 (5.56)	1.00(0.96–1.05)	0.851
	Warmth (paternal)	15.04 (5.35)	15.47 (5.51)	0.99(0.94–1.03)	0.556
	Authoritarianism (maternal)	8.78 (3.76)	9.14 (3.87)	0.98(0.91–1.04)	0.451
	Authoritarianism (paternal)	9.06(3.85)	9.16 (3.27)	0.99(0.92–1.06)	0.762
	Protectiveness (maternal)	10.48 (3.27)	9.84 (3.04)	1.07(0.99–1.16)	0.112
	Protectiveness (paternal)	9.96(3.22)	9.42(2.83)	1.06(0.97–1.15)	0.184

## Discussion

In our survey of 5,996 women with recurrent MDD we found that 7% reported ever having smoked and 6% were lifetime regular smokers. Smokers were slightly younger and were almost three times more likely to be unmarried. Comparing those who had smoked (lifetime smokers) to non-smokers we found a number of clinical features and risk factors that differentiated the two groups. Smokers had a slightly more severe illness, as characterized by more episodes, fulfilling more symptomatic DSM-IV criteria and more comorbid anxiety (particularly panic disorder). Smokers were more neurotic, reporting more stressful life events, and more perceived protective parenting. They were also more likely to report a family history of MDD. Nicotine dependent smokers (those with FTND scores greater than 5) were significantly more neurotic than other smokers. We comment on these issues below.

The smoking rates we report are similar to those found in surveys of Chinese women in the PRC. In 1996 Yang et al recruited 56,020 female participants in China and the prevalence rate for smokers was 4.2% [2]. Ma et al recruited 3191 women in Beijing of China in 2009, and reported the prevalence of life time smoking to be 6.9%. [27]. World Health Organization reported a prevalence rate for current smoking of 2.4% in Chinese women in 2011 [28]. In 2012 Giovino GA et al reported smoking prevalence in Chinese women was 2.4% [29]. Altogether, the smoking rates (current or lifetime smoking) remain at very low levels (2.4%–6.9%).

Compared to other countries where the relationship between depression and smoking has been assessed, smoking rates for women in China are relatively low [17], [30], [31], [32]. The relatively low prevalence of smoking in our depressed women is likely due to cultural factors [33]. Female smoking rates are very low in China and South Korea, where Confucianism and patriarchy still strongly limit women's sociopolitical status [2], [34]. Gender roles and social norms may protect women from smoking [35].

We found that association between smoking and depression is mediated by common familial and environmental factors, as others have found [5], [11], [36], [37], [38], [39], [40], [41], though in our study design we cannot distinguish which factors are causal. We found that socioeconomic status makes a relatively small contribution to the increased rates of smoking among depressed women. Many studies in the West have shown that the smoking prevalence rate is higher among those in lower socioeconomic groups [42], [43], [44]. Such studies took educational level, occupation, income, ownership of estate and marital status to evaluate socioeconomic status. Most of the research found that lower income, poor education, lower educational level and abnormal marital status are related to smoking [45], [46], [47]. It is suggested that the lower socioeconomic status may induce stress and the stress leads to smoking [48].

The relatively small impact of socio-economic factors in our study is likely explicable by the fact that China remains a male-dominated society. After marriage most women resign from work and pay much more attention to house-keeping and baby care. Therefore most of the family income depends on their husbands [49]. This means that the socio-economic status of the family is determined mainly by the male's occupation and social class, not the female's. The females' perceived economic situation is affected mainly by their husbands. However this kind of perception is associated with smoking [50].

The relatively large increase in risk of smoking in unmarried women may have a cultural explanation. In Chinese culture unmarried women feel ashamed, especially those who have never married, who have separated or divorced. These women may experience greater feelings of isolation from cultural norms, and this may encourage them to take up smoking. Second, married women are less likely to smoke because many of them take responsibility for house-keeping and child-care [49]. Smoking is regarded as unhealthy for children, and is restricted by other family members. In contrast, single, divorced, or widowed women are relatively free from these social limitations, and are therefore more likely to engage in smoking.

Our study is a cross-sectional one so we are unable to determine whether smoking increased the risk of MDD or vice versa. We found some evidence for a causal role of smoking, in that more patients reported starting smoking before the onset of MDD, but this is not a strong effect. Some cross-sectional studies show that those who smoke suffer more depressive and anxiety symptoms [51], [52], [53], [54]. Other studies report that nicotine dependent smokers experience more severe depressive and anxiety symptoms than those without nicotine dependence. [55], [56], [57] We were not able to examine this hypothesis. The rates of smoking were not assessed in controls so we could not examine the association between MDD and smoking.

A direct causal mechanism linking smoking to MDD is suggested by findings that smoking affects reward pathways in the brain that have been related to changes in mood. Both smoking and depression alter levels of dopamine [58], [59], brain-derived neurotrophic factor (BDNF) levels [60], [61], and alter the activity of monoamine oxidase (MAO) [62], [63]. Future research should focus more on how smoking affects the concentration of DA and BDNF and the activity of MAO and how smoking together with depression affect the neurotransmission system mentioned above.

A number of studies have found association between nicotine dependence and neuroticism [64], [65], [66] and anxiety disorders [9], [67]. We found convincing evidence that increased neuroticism scores are related to nicotine dependence. We did not detect environmental risk factors significantly associated with ND (which others have found [64], [65]), nor did association with anxiety reach statistical significance.

The results of our study are subject to a number of limitations. First, the cases were ascertained in a clinical setting, and so are not representative of community samples; second, all cases are Han Chinese women, consequently results may not apply to males or to other ethnicities; third the cross-sectional design of the study precludes inferences about the causal relation between smoking and MDD; fourth, without a biological marker of smoking behavior, we can not establish to what extent reported smoking represents true smoking. Since smoking is a deviant behavior in China, some of our findings may be due to a confound between the tested variables and willingness to report smoking. Finally, our sample size is relatively small, and will limit our power to detect any weak, but potentially important, associations.
